# John L. Markley, a legacy of service in structural biology

**DOI:** 10.1063/4.0001229

**Published:** 2026-09-08

**Authors:** Jeffrey C. Hoch, Stephen K. Burley, Genji Kurisu, Kyle L. Morris, Sameer Velankar

**Affiliations:** 1 Biological Magnetic Resonance Data Bank, Department of Molecular Biology and Biophysics, University of Connecticut, Farmington, Connecticut 06030-3305, USA; 2 Research Collaboratory for Structural Bioinformatics Protein Data Bank and the Institute for Quantitative Biomedicine, Rutgers, The State University of New Jersey, Piscataway, New Jersey 08854, USA; 3 Rutgers Cancer Institute, Rutgers, The State University of New Jersey, New Brunswick, New Jersey 08901, USA; 4 Department of Chemistry and Chemical Biology, Rutgers, The State University of New Jersey, Piscataway, New Jersey 08854, USA; 5 Rutgers Artificial Intelligence and Data Science (RAD) Collaboratory, Rutgers, The State University of New Jersey, Piscataway, New Jersey 08854, USA; 6 Research Collaboratory for Structural Bioinformatics Protein Data Bank, San Diego Supercomputer Center, University of California, San Diego, La Jolla, California 92093, USA; 7 Protein Data Bank Japan, Institute for Protein Research, The University of Osaka, Suita, Osaka 565-0871, Japan; 8 Protein Data Bank Japan, Protein Research Foundation, Minoh, Osaka 565-8686, Japan; 9 Electron Microscopy Data Bank, European Molecular Biology Laboratory, European Bioinformatics Institute, Hinxton, Cambridge CB10 1SD, United Kingdom; 10 Protein Data Bank in Europe, European Molecular Biology Laboratory, European Bioinformatics Institute, Hinxton, Cambridge CB10 1SD, United Kingdom

## Abstract

John L. Markley was an early pioneer of the application of nuclear magnetic resonance (NMR) spectroscopy to investigate the structure, dynamics, and function of biological macromolecules by NMR. A recurring theme of his career is the development of standards and shared resources that have enabled the biological NMR community to coherently and collectively advance the disciplines of structural biology and metabolomics. Spanning development standards for atom nomenclature, founding shared NMR resources at Purdue and UW-Madison, and founding the Biological Magnetic Resonance Data Bank (BMRB) and shepherding BMRB membership in the Worldwide Protein Data Bank, this appreciation revisits the many and lasting contributions by our friend and colleague John Markley in support of the global structural biology community.

## INTRODUCTION

From the very start of his graduate studies with Professor Oleg Jardetzky at Harvard Medical School in the fall of 1964, John Markley was a pioneer of biomolecular nuclear magnetic resonance (NMR). Saunders, Wishnia, and Kirkwood had demonstrated the 40 MHz proton NMR spectrum in 1957.^1^ By 1964 magnetic field strengths had increased to 100 MHz proton frequencies, and it was already understood that folded and denatured proteins yielded distinctly different spectra, with the latter resembling the superimposed spectra of the constituent amino acids. Moreover, it was appreciated that resolved NH resonances offered the potential for both atomic-level insights in three dimensions (3D) and the influence of pH on protein activity. Once Jardetzky moved to the Merck Institute, Markley had access to a state-of-the-art 100 MHz spectrometer. In his first publications with Jardetzky, Markley exploited global characteristics of ^1^H spectra of proteins to characterize helix-coil transitions in synthetic poly-amino acids and well-resolved histidine resonances to investigate conformational change in the enzyme staphylococcal nuclease.^2^ Soon thereafter, he adopted the strategy of selective isotope labeling to obtain atomic-level insights from individual amino acids in proteins that were otherwise inaccessible from one-dimensional spectra at the limited resolution afforded by magnets then available. In 1968, he published a groundbreaking investigation on selectively deuterated staphylococcus nuclease.^3^ It is our conjecture that the need for precision in describing the location of stable isotopes fostered in Markley an early appreciation for the importance of clear and consistent community-agreed data standards and his commitment to broader community-based initiatives that would become a defining characteristic of his career.

Markley's scientific contributions, notably stable isotope labeling for bioNMR and studies on paramagnetic proteins and low-barrier hydrogen bonds, have been ably and extensively reviewed by others. The focus of this tribute is on his many contributions to the broader biological NMR and structural biology communities.

## BIOLOGICAL MAGNETIC RESONANCE DATA BANK

The first protein structure determined by NMR was reported by Wuthrich and coworkers in 1985.^4^ Sequential assignments had already been reported for a number of proteins, and it was clear that NMR could be used to determine 3D structures of biomolecules, the only method capable of doing so in solution. The Protein Data Bank was established in 1971,^5^ and in 1989, when the first two NMR protein structures were deposited, the total number of structures archived in the PDB stood at less than 100 (89 to be precise). NMR consistently contributed more than 25% of structured deposits to the PDB annually through 2005, with the total number of NMR structures per year peaking at 965 in 2007. Markley and Eldon Ulrich realized the need in the late 1980s for a shared data resource hosting NMR primary data used to make sequential resonance assignments and determine biomolecular structures. In 1988, they received an NIH grant to establish the Biological Magnetic Resonance Data Bank (BMRB). In 2002, BMRB Japan (BMRBj, formerly branded as PDBj-BMRB) was established at Osaka University. BMRBj currently processes BMRB depositions coming from Asia and Oceania. In 2006, a BMRB mirror site (providing data-out services only) was established at the Centro di Resonanze Magnetiche (CERM) in Florence, Italy, headed by the late Ivano Bertini. Today BMRB contains millions of assigned chemical shifts, many thousands of relaxation rates for biomacromolecules, and copious NMR data for small molecules of biological interest.

## COMMUNITY DATA STANDARDS

A critically important step, essential for comparative analysis of chemical shifts in biomacromolecules and for establishing structural correlations, is establishing a common referencing standard. Nuclear resonance frequencies are measured with a precision exceeding 1 part in 10^9^, and field variations due to intramolecular effects span 1 part in 10^7^ for protons, with greater variations observed for some nuclei (e.g., ^19^F). Slight differences in the polarizing magnetic field produced by the instrument due to shim currents and other instrumental effects can create much larger variations, even on the same instrument. Correcting for these differences requires the use of an atomic reference, typically a compound added to the sample or isolated from the sample via a coaxial capillary. Markley was a coauthor in 1995 on a report that established widely adopted protocols for chemical shift referencing in biomolecular NMR.^6^ Equally important for comparative analysis of biomolecular structures determined by NMR is standard nomenclature for describing biomolecular structures determined by NMR. Markley led a community-based effort to establish standard nomenclature, publishing recommendations in 1998.^7^ BMRB was a key player in both initiatives. Without these standards, accumulating biomolecular NMR data in an open data resource would have offered little value.

## PURDUE UNIVERSITY BIOLOGICAL MAGNETIC RESONANCE LABORATORY

Markley was appointed to the Purdue University faculty in 1972. In 1977, he was awarded an NIH P41 Grant to establish the Purdue University Biological Magnetic Resonance Laboratory (PUBML), one of the first regional resources for NMR spectroscopy (joining facilities at Carnegie-Mellon, The Rockefeller University, Yale University, and the University of Illinois).

## INTERNATIONAL CONFERENCE ON MAGNETIC RESONANCE IN BIOLOGICAL SYSTEMS

Markley attended the second International Conference on Magnetic Resonance in Biological Systems (ICMRBS) in 1996, held in Stockholm. The first ICMRBS meeting was organized by his mentor, Jardetzky, together with Robert Shulman and Mildren Cohn. In 1984, Markley was elected to the ICMRBS Council. In 1988, he hosted the ICMRBS meeting in Madison, Wisconsin as Conference Chair. From 2016 to 2018, he served as ICMRBS Treasurer.

### NMR Facility at Madison

In 1985, Markley moved to the University of Wisconsin-Madison. In 1985, he was again awarded an NIH P41 Grant, this time to establish the NMR Facility at Madison (NMRFAM). Over time NMRFAM grew to become a national resource, which currently operates 13 NMR spectrometers ranging in field strength from 400 MHz to 1.1 GHz, supporting both liquid- and solid-state experiments. In just the last 5 years, NMRFAM supported more than 193 labs based in 34 states. Dozens of junior faculty earned tenure because of experiments they were able to conduct at NMRFAM. Quite a remarkable legacy in and of itself.

## CENTER FOR EUKARYOTIC STRUCTURAL GENOMICS

In 2001, Markley was awarded an NIH Grant as part of the NIGMS Protein Structure Initiative to establish the Center for Eukaryotic Structural Genomics (CESG) at the University of Wisconsin-Madison. During his more than decade-long tenure as director of the Center, CESG developed and disseminated technologies for high-throughput protein purification and expression and important software tools, including the Sesame Laboratory Information Management System (LIMS) and PINE-NMR for analyzing NMR-derived protein structures. CESG is credited with depositing 219 protein structures to the PDB.

### Worldwide Protein Data Bank

The PDB was founded in 1971 at Brookhaven National Laboratory. In 1998, stewardship of PDB transferred to the RCSB, a consortium consisting of groups at Rutgers, SDSC, and NIST. The Worldwide Protein Data Bank (wwPDB) was founded in 2003 by the RCSB Protein Data Bank, the Macromolecular Structure Databank (now Protein Data Bank in Europe), and Protein Data Bank Japan. Under Markley's leadership, BMRB completed the requirements for joining the wwPDB^8^ in 2006.

## NETWORK FOR ADVANCED NMR

Nearing retirement, Markley continued to contribute to community-based initiatives, including a novel collaboration among UW-Madison and the Universities of Georgia and Connecticut to create the Network for Advanced NMR (NAN). He contributed to the NSF Midscale RI-2 proposal that launched NAN in 2022. NAN has the broad goal of further democratizing access to high-field NMR instrumentation for applications across the sciences, including material sciences and metabolomics in addition to structural biology. NAN installed and commissioned the first two open-access 1.1 GHz NMR instruments in the US, for solid-state applications at NMRFAM and for solution-state applications at the Complex Carbohydrate Research Center at the University of Georgia. Though Markley retired prior to the official announcement of the NSF Midscale Grant award, NAN continues to embody many of the principles of community service that characterized his long and enormously impactful career.

## CONCLUDING REMARKS

John Markley's contributions to community standards and resources in structural biology and biomolecular NMR spectroscopy represent an enduring legacy benefitting the current generation and supporting future contributors to biomolecular NMR and metabolomics.

## Data Availability

The data that support the findings of this study are available from the corresponding author upon reasonable request.
